# Supplementary material for: Identification of photoperiod-induced specific miRNAs in the adrenal glands of Sunite sheep (Ovis aries)
Source: Front Vet Sci. 2022 Jul 22;9:888207. doi: 10.3389/fvets.2022.888207 (PMC9354845; doi:10.3389/fvets.2022.888207)
Supplement: Supplementary file 1 [file Data_Sheet_1.ZIP › Supplementary file/miRDeep2 file.zip!/miRDeep2 file/Know_Structure/LP42A3/oar-mir-200a.pdf]

miRBase precursor : oar-mir-200a
Total read count : 95
oar-miR-200a read count : 93
remaining reads : 2

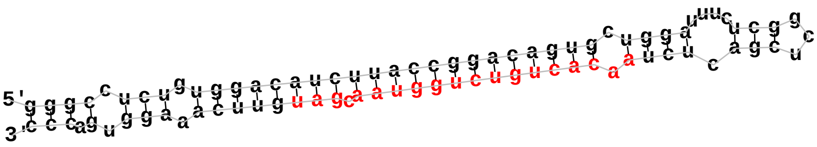

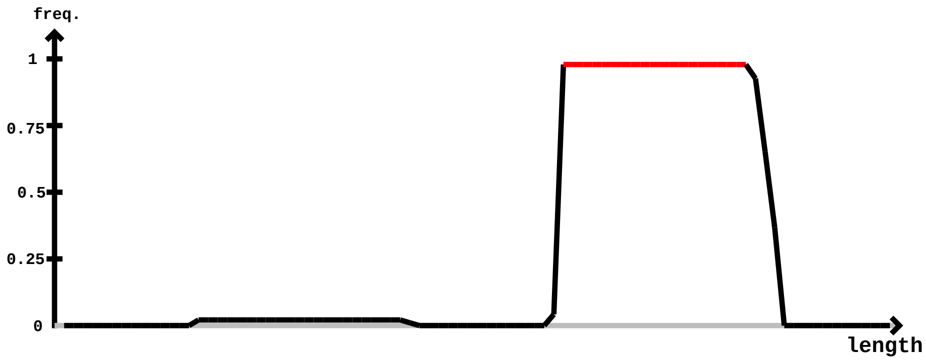

oar-miR-200a

```
5'- gggccucuguggacaucuuaccggacagugcuggauuucucggcucgacucuaacacugucugguaacgauguucaaaggugaccc   -3'    exp
    ((((.(((.((((((((((((((((((((.((((....(((...))).)))).))))))))))))).)))))))).))).).)))   reads      mm         sample
    .............caucuuaccggacagugcugga...................................................   1          0          seq
    .............caucuuaccggacagugcuggau..................................................   1          0          seq
    ..................................................cuaacacugucugguaaAgaug..............   4          1          seq
    ...................................................uaacacugucugguaaAgau...............   5          1          seq
    ...................................................uaacacugucugguaacgaug..............   10         0          seq
    ...................................................uaacacugucugguaaAgaug..............   12         1          seq
    ...................................................uaacacugucugguaacgaugu.............   27         0          seq
    ...................................................uaacacugucugguaacgauguu............   35         0          seq
```
